# Supplementary material for: Classification of images using Gaussian copula model in empirical cumulative distribution function space
Source: PLoS One. 2024 Dec 6;19(12):e0309884. doi: 10.1371/journal.pone.0309884 (PMC11623484; doi:10.1371/journal.pone.0309884)
Supplement: Attachment — Submitted filename: Review_copula_2.pdf [file pone.0309884.s002.pdf]

Second Review on "Classification of images using Gaussian copula model in empirical cumulative distribution function space"

The authors have resolved some issues I raised in the last review. However, I regret to note that there are still many errors, both in the formulas and in the writing.

First of all, in line 99, the index u of $X_{j,u}$ should be i. In line 131, the letter H is missing, and the index of y should be reviewed because $y_{p,s}$ should be y_{kps}. In line 139, the partial derivative expression in the numerator should not have the superscripts ps. There are other oversights regarding the indexes, which should be reconsidered for mathematical rigor. Please reread and correct all oversights and inaccuracies throughout the manuscript.

An important issue, Figure 3 represents the quantile function and not the empirical cumulative distribution function (ECDF). Additionally, $\tilde{x}$ must be written correctly and described as $\mathrm{vec}(\tilde{A})$, because otherwise it is not clear for a reader. It is important to note that the graph shows the quantile function, not the ECDF. Consequently, the graphs in Figure 4 also correspond to quantile functions.

Figure 5 indicates the quantile function on the side, with the probability density at the bottom and the corresponding cumulative distribution. The letter G indicating the density should be lowercase if the uppercase G indicates the cumulative distribution. These oversights must to be corrected.

In Table 2, the parameters for the beta distribution should be labeled as "beta distribution parameters" instead of "beta parameters."

Overall, the idea is original and deserves to be published. However, there are serious mathematical inaccuracies that need to be carefully reviewed. The topic of copulas is not trivial, and I understand that adapting the formulas for the specific case is very difficult. Once all these issues are addressed, I can reconsider the manuscript for publication.
