# Supplementary material for: Classification of images using Gaussian copula model in empirical cumulative distribution function space
Source: PLoS One. 2024 Dec 6;19(12):e0309884. doi: 10.1371/journal.pone.0309884 (PMC11623484; doi:10.1371/journal.pone.0309884)
Supplement: Attachment — Submitted filename: #PONE-D-23-26454-Response to reviewers-round 2.pdf [file pone.0309884.s003.pdf]

PONE-D-23-26454
Classification of images using Gaussian copula model in empirical cumulative distribution function space
PLOS ONE

Reviewer Comments:

1. *Comment: "In line 99, the index u of $X_{j,u}$ should be i. In line 131, the letter H is missing, and the index of y should be reviewed because y_{ps} should be y_{kps}. In line 139, the partial derivative expression in the numerator should not have the superscripts ps. There are other oversights regarding the indexes, which should be reconsidered for mathematical rigor. Please reread and correct all oversights and inaccuracies throughout the manuscript.Authors have addressed the queries raised by the reviewer. But, some typo errors are seen through out the paper. The manuscript need to be thoroughly checked."*

   Response:

   Thank you for your valuable feedback. We have reviewed and corrected all the writing errors you mentioned.

   Line 99 : The index u of $X_{j,u}$ has been changed to $X_{j,i}$ (line 100).

   Line 131 : The missing letter H has been added, and the index of for y has been corrected from y_{ps} to y^{p}_{k,n_p} (line 130).

   Line 139 : The partial derivative expression in the numerator has been corrected by removing the superscript ps (line 140).

   Additionally, we have revised the notation for the indices, moving the indices for partitioning from below to above to facilitate better understanding of the model. For example, the notation $y_{k,j,t}$ which is a random variable on class k, partition j and distinguishing point t is changed to $y^{j}_{k,t}$. Then for the number of distinguishing points in each partition which was originally denoted by s the writing was changed to a more general np, adjusting to the number of distinguishing points in the partition.

   We have conducted a thorough review to ensure that there are no other notation and indexing errors throughout the manuscript. All inaccuracies and oversights have been corrected to

ensure mathematical rigor. Once again, thank you for your helpful suggestions and comments.

2. *Comment: "An important issue, Figure 3 represents the quantile function and not the empirical cumulative distribution function (ECDF). Additionally,* $\tilde{x}$ *must be written correctly and described as* $vec(\widetilde{A})$*, because otherwise it is not clear for a reader. It is important to note that the graph shows the quantile function, not the ECDF. Consequently, the graphs in Figure 4 also correspond to quantile functions.A block diagram is needed for better understanding by the reader."*

Response:

Thank you for your valuable feedback. We appreciate the clarity you expect regarding Figure 3 and Figure 4 in our manuscript. We would like to clarify that Figure 3 actually represents the Empirical Cumulative Distribution Function (ECDF), not the quantile function as mentioned. ECDF is an appropriate method to describe the cumulative distribution of pixel values that have been normalized in the range [0, 1]. We will update the paragraph on line 351 to make it clearer.

> The elements of the data matrix $\boldsymbol{A}$ have components x, which is a representation of the pixel intensity values. We convert each x element of the data matrix, initially in the range $[0,255]$, to the range $[0,1]$ by dividing it by 255, expressed as $\tilde{x} = x/255$. This transformation results in $\tilde{x}$ values that are easy to handle in numerical calculations, especially in machine learning algorithms and statistical analyses, which are more stable and fast when performed in the interval $[0,1]$. When the vectorization process converts the data matrix A into a vector, the result is the vector $Vec(\boldsymbol{A})$. Moreover, the transformation of matrix $\boldsymbol{A}$ yields the matrix $\widetilde{\boldsymbol{A}}$, which subsequently transforms into the vector $Vec(\widetilde{\boldsymbol{A}})$. Next, the ECDF is constructed by following the method specified in Eq (1). Fig 3 visually illustrates the construction of the ECDF. The ECDF serves as an image characteristic, capturing the pixel values that are less than or equal to x. These characteristics show variations in different image classes, underscoring the importance of identifying specific realization points of x that can effectively distinguish them.

I apologize for any inconvenience, but the notation $\tilde{x}$ in the plots cannot be corrected without rerunning the MATLAB code since the images are not saved.

3. *Comment: "Figure 5 indicates the quantile function on the side, with the probability density at the bottom and the corresponding cumulative distribution. The letter G indicating the density should be lowercase if the uppercase G indicates the cumulative distribution. These oversights must to be corrected.More explanation is required for the results and discussion."*

   Response:

   Thank you for your comments and observations. We will address the following points:

   a. Figure 5 Clarification: We will ensure that the letter indicating density g is lowercase, while G will denote the cumulative distribution function (CDF). This correction will be implemented to accurately represent both the quantile function and the corresponding probability density.

   b. Additional Explanation in Results and Discussion: We recognize the need for more detailed explanation and elaboration in the results and discussion sections. We will provide further clarity to ensure thorough understanding of the presented findings and their implications.

   We appreciate your feedback, which will help us enhance the clarity and accuracy of our manuscript.

4. *Comment: "In Table 2, the parameters for the beta distribution should be labeled as " beta distribution parameters" instead of " beta parameters.""*

   Response:

   Thank you for your comment. We have updated Table 2 to label the parameters for the beta distribution as "beta distribution parameters" instead of "beta parameters" to ensure clarity and accuracy.

5. *Comment: "Overall, the idea is original and deserves to be published. However, there are serious mathematical inaccuracies that need to be carefully reviewed. The topic of copulas is not trivial, and I understand that adapting the formulas for the specific case is very difficult. Once all these issues are addressed, I can reconsider the manuscript for publication."*

   Response:

Thank you for your thoughtful review and feedback. We appreciate your recognition of the originality of our work and your consideration for publication. We recognize the importance of addressing mathematical inaccuracies and ensuring the correctness of formulas, especially in adapting them for the specific case of copulas.

We are committed to thoroughly reviewing and correcting all identified issues to meet the standards required for publication. Your comments will guide us in making the necessary improvements, and we look forward to resubmitting the manuscript for your reconsideration once the revisions are completed.

We commit to revising the manuscript in accordance with these valuable suggestions and feedback. Thank you for your time and consideration.

Best regards,

Dr. Sri Winarni

Department of Statistics, Faculty of Mathematics and Natural Sciences, Padjadjaran University, West Java, Indonesia
